# Data-driven identification of plasma metabolite clusters and metabolites of interest for potential detection of early-stage non-small cell lung cancer cases versus cancer-free controls

**DOI:** 10.1186/s40170-022-00294-9

**Published:** 2022-10-12

**Authors:** Julian O. Kim, Robert Balshaw, Connel Trevena, Shantanu Banerji, Leigh Murphy, David Dawe, Lawrence Tan, Sadeesh Srinathan, Gordon Buduhan, Biniam Kidane, Gefei Qing, Michael Domaratzki, Michel Aliani

**Affiliations:** 1 Section of Radiation Oncology, Department of Radiology, Max Rady Faculty of Health Sciences, University of Manitoba, Winnipeg, Manitoba Canada; 2 CancerCare Manitoba Research Institute, Winnipeg, Manitoba Canada; 3 George and Fay Yee Center for Healthcare Innovation, University of Manitoba, Winnipeg, Manitoba Canada; 4 Department of Computer Science, University of Manitoba, Winnipeg, Manitoba Canada; 5 Section of Medical Oncology, Department of Internal Medicine, Max Rady Faculty of Health Sciences, University of Manitoba, Winnipeg, Manitoba Canada; 6 Department of Biochemistry and Medical Genetics, University of Manitoba, Winnipeg, Manitoba Canada; 7 Section of Thoracic Surgery, Department of Surgery, Max Rady Faculty of Health Sciences, University of Manitoba, Winnipeg, Manitoba Canada; 8 Department of Human Pathology, Max Rady Faculty of Health Sciences, University of Manitoba, Winnipeg, Manitoba Canada; 9 Food and Human Nutritional Sciences, University of Manitoba, Winnipeg, Manitoba Canada

**Keywords:** Early-stage non-small cell lung cancer, Plasma metabolomics, Early detection, Non-targeted metabolomics

## Abstract

**Background:**

Metabolomics is a potential means for biofluid-based lung cancer detection. We conducted a non-targeted, data-driven assessment of plasma from early-stage non-small cell lung cancer (ES-NSCLC) cases versus cancer-free controls (CFC) to explore and identify the classes of metabolites for further targeted metabolomics biomarker development.

**Methods:**

Plasma from 250 ES-NSCLC cases and 250 CFCs underwent ultra-high-performance liquid chromatography/quadrupole time-of-flight mass spectrometry (UHPLC-QTOF-MS) in positive and negative electrospray ionization (ESI) modes. Molecular feature extraction, formula generation, and find-by-ion tools annotated metabolic entities. Analysis was restricted to endogenous metabolites present in ≥ 80% of samples. Unsupervised hierarchical cluster analysis identified clusters of metabolites. The metabolites with the strongest correlation with the principal component of each cluster were included in logistic regression modeling to assess discriminatory performance with and without adjustment for clinical covariates.

**Results:**

A total of 1900 UHPLC-QTOF-MS assessments identified 1667 and 2032 endogenous metabolites in the ESI-positive and ESI-negative modes, respectively. After data filtration, 676 metabolites remained, and 12 clusters of metabolites were identified from each ESI mode. Multivariable logistic regression using the representative metabolite from each cluster revealed effective classification of cases from controls with overall diagnostic accuracy of 91% (ESI positive) and 94% (ESI negative). Metabolites of interest identified for further targeted analysis include the following: 1b, 3a, 12a-trihydroxy-5b-cholanoic acid, pyridoxamine 5′-phosphate, sphinganine 1-phosphate, gamma-CEHC, 20-carboxy-leukotriene B4, isodesmosine, and 18-hydroxycortisol.

**Conclusions:**

Plasma-based metabolomic detection of early-stage NSCLC appears feasible. Further metabolomics studies targeting phospholipid, steroid, and fatty acid metabolism are warranted to further develop noninvasive metabolomics-based detection of early-stage NSCLC.

**Supplementary Information:**

The online version contains supplementary material available at 10.1186/s40170-022-00294-9.

## Background

Lung cancer is the leading cause of cancer-related mortality worldwide with an estimated 1.59 million persons succumbing to lung cancer annually [1]. Presently, most lung cancers are detected at either locally advanced or metastatic stages for which chances of cure are limited. Earlier detection of lung cancer represents a strategy to reduce mortality by allowing more patient opportunities for curative therapies including surgery or stereotactic ablative radiotherapy before disease progression to incurable disease. As proof of this principle, several randomized trials [2] have demonstrated that early detection of lung cancer using low-dose CT screening scans reduces lung cancer mortality. Although CT screening is being adopted in various jurisdictions, concerns remain regarding financial burden [3], high false-positive rate which can trigger unnecessary ancillary test procedures [4, 5], and risks of radiation-induced malignancy [6, 7]. Thus, there remains a bona fide need for the development of a cost-effective, noninvasive, accurate test for early NSCLC detection.

Metabolomics is an omics field which consists of measuring endogenous and exogenous low-molecular-weight metabolites in an organism at a specified time under specific environmental conditions [8]. Alterations in the metabolomic phenotype of cancer cells were first reported in 1956 by Warburg et al. who observed a higher rate of glycolysis and lactic acid production [9]. Technological advancements over the subsequent decades have led to the use of metabolomics methods including gas chromatography (GC), mass spectrometry (MS), liquid chromatography (LC), and ^1^H-nuclear magnetic resonance (^1^H-NMR) as means for the diagnosis and prediction of outcomes in oncology. Metabolomic profiling has been used to assess the impact of malignancy on the metabolome using tumor tissue [10], urine [11], blood [12, 13], stool [14], or other biofluids [15]. To date, several groups [12, 13, 16–22] have investigated metabolomics profiles of NSCLC cases versus controls using combinations of different analytic platforms, biofluids, patient populations, and statistical modeling approaches.

With variations in methodologies employed in these studies, there is minimal overlap of metabolomic profiles that distinguish non-small cell lung cancer (NSCLC) cases from controls. In addition to the differences in analytical approaches, the lack of assessment for the impact of potential confounding clinical variables such as age, sex, or smoking status may have contributed to this lack of similarity. Furthermore, these studies utilized relatively modest numbers of NSCLC cases for analysis and profile training, and few have focused primarily on the detection of early-stage NSCLC cases (ES-NSCLC), the group for which early detection is most relevant and technically challenging.

This metabolomics biomarker discovery study assessed the differences in global metabolomics profiles of plasma from a large group of clinical ES-NSCLC patients versus cancer-free controls to reduce the risk of model overfitting. We aimed to determine which classes of metabolites appear promising for future use in targeted metabolomics studies to further refine metabolomics profiles associated with ES-NSCLC. Our secondary aim was to assess the impact of clinical covariates, namely age, smoking history, and sex, on the classification performance of metabolomic entities for differentiating ES-NSCLC cases from cancer-free controls.

## Methods

### Patient population

From 2004 to 2014, 250 patients with clinical early-stage lung cancer (based on preoperative CT imaging scans) had blood samples collected by venipuncture prior to surgical resection. Final pathological staging was assigned following pathological analysis of pulmonary resection specimens using AJCC 6th edition. Blood samples were centrifuged into component blood products, and plasma aliquots were frozen at −80 °C and stored at the Manitoba Tumour Bank (MTB), a provincial biorepository certified by the Canadian Tissue Repository Network. Surgery for NSCLC cases consisted of wedge resection, segmentectomy, or lobectomy with lymph node sampling or lymph node dissection as clinically indicated. During the same time period, plasma samples from cancer-free controls were collected by venipuncture and stored using identical laboratory procedures in the same biorepository. Cancer-free controls consisted of two groups: (1) patients with suspected lung cancer (based on preoperative imaging) who underwent surgical resection with final pathology showing only benign pulmonary disease such as tuberculosis, granulomatous disease, or pulmonary fibrosis and no evidence of malignancy and (2) family members of cancer patients with no prior personal history of malignancy (with exception of completely excised non-melanomatous skin cancers). Controls were excluded if cancer was diagnosed within 2 years following the date of the blood collection. Fasting was not mandatory prior to blood collection, and so fasting and non-fasting specimens were included in the analysis.

### Metabolomics analysis

All analytical work was carried out in duplicate by blinded laboratory personnel who are unaware of the case/control status of plasma samples so to reduce unintentional bias. The laboratory workflow and procedures employed have been previously published [23, 24] and are summarized briefly below.

#### Plasma extraction

Frozen aliquots of plasma were thawed to 20 °C, and four 100-μL aliquots were extracted per sample. For each aliquot, 200 μL of acetonitrile was added and vortexed for 30 s and centrifuged at 10,000*g* for 10 min at 4 °C, and then, 250 μL of supernatant was pipetted into an Eppendorf tube and dried under a gentle stream of N_2_ and stored at −80 °C. The dried samples were reconstituted in 100 μL of 4:1 acetonitrile and deionized H_2_O prior to analysis.

#### Ultra-high-performance liquid chromatography/quadrupole time-of-flight mass spectrometry (UHPLC-QTOF-MS) analysis

A 1260 Rapid Resolution system (Agilent Technologies, Santa Clara, USA) containing a binary pump and degasser, well-plate autosampler, and thermostatted column compartment (maintained at 55 °C) was used for analyses. Chromatographic separations were performed in duplicate on an Agilent ZORBAX SB-Poroshell column 2.1 mm × 50 mm, 2.7 μm. MS analysis was performed on an Agilent 6538 QTOF mass spectrometer equipped with dual electrospray ionization (ESI) source in positive and negative modes.

#### Identification of metabolic entities

Agilent Profinder software, version B.08 (Agilent Technologies, Santa Clara, USA) including molecular feature extraction (MFE), formula generation, and find-by-ion tools, was used to prepare raw data and identify individual metabolic entities. Log2 normalization of the concentrations of individual metabolites was performed using the Mass Profiler Professional software, version 12.6 (Agilent Technologies, Santa Clara, USA), and exported as raw data files for model building.

#### Quality control

Five quality control mixtures were made by pooling 100 μL of plasma (randomly chosen from 10 samples in each group) and were analyzed in a random manner amongst all other samples.

### Statistical analysis

Patient characteristics recorded at the time of sample collection were tabulated by case versus control status and compared using the Welch two-sample *t*-test or Pearson’s chi-squared test.

#### Data filtration and data cleaning

Data analysis was restricted to known endogenous human metabolites identified by the Metlin and Human Metabolome databases. Candidate endogenous metabolites not detected in 80% or more of all samples were judged to be unlikely to possess useful classification values and were dropped from further analysis. Missing values of individual metabolites were replaced with one-half of the smallest positive measured quantity for each metabolite. All data analysis was conducted using the R statistical software package.

#### Cluster analysis

Given the highly dimensional nature of metabolomics data and the apparent risk of numerous collinear entities to mask overall global alterations present in the data, an unsupervised hierarchical cluster analysis was used to abridge the data into groups of 12 subsets of similar metabolites based on their distance in multivector space (1-correlation) using the complete linkage method of the Log2 normalized concentrations for each metabolite, and samples were clustered using the Mahalanobis distance using complete linkage method. For the cluster analyses, the averaged value of the Log2 normalized concentrations of metabolites arising from duplicated UHPLC-QTOF-MS assessments was used, and separate cluster analyses were done for the ESI positive and negative ionization modes. Hierarchical cluster analysis dendrogram heat maps were generated to display clusters of similar metabolites with individual metabolites on the *x*-axis and individual samples on the *y*-axis. A principal component analysis (PCA) of the cohort was performed to visually and qualitatively assess the separation of cases from controls in multivector space by ESI mode using 12 cluster-representative metabolic entities per ESI mode. For this PCA, the metabolite within each cluster with the strongest correlation to the first principle component was designated a “cluster-representative metabolite,” which was tabulated by ESI mode to provide qualitative examples of entities within each cluster.

#### Logistical regression modeling

To assess the classification potential of metabolomic entities in determining case versus control status, the 12 cluster-representative metabolites identified from ESI-positive and ESI-negative cluster analyses were utilized as explanatory variables in a multivariable logistic regression model for the endpoint of NSCLC case status, with a separate logistic regression model for the ESI-positive and ESI-negative modes. With the aim of exploring the impact of potential clinical confounding variables (known lung cancer risk factors) on model fit, two additional multivariable logistic regression models were built which included clinical explanatory variables (age, sex, and smoking history) in addition to the 12 cluster-representative metabolites for each ESI mode. Forest plots were generated to visualize the relative strength of association and distribution of odds ratios associated with each cluster-representative metabolite with and without adjustment for clinical explanatory variables. Volcano plots were generated of cluster-representative metabolites from each ESI mode to visualize the negative log *p*-values (−log_10_ (corrected *p*-value)) from Welch’s *t*-test (unadjusted *p*-values) versus the Log_2_ fold change in the mean concentration between cases and controls for each metabolite.

#### Classification performance

Classification performance of cluster-representative metabolites was assessed by multiplying regression coefficients for every 12 representative metabolites by the Log2 normalized concentrations of the same metabolites for each patient and comparing logistic regression predicted case versus control status versus known case versus control identity. Sensitivity, specificity, and overall classification accuracy by ESI mode both with and without the inclusion of clinical covariates were calculated, and receiver operator characteristic (ROC) curves were generated plotting sensitivity as a function of (1-specificity). The workflow used for this study is summarized in supplemental Table A1.

### Ethical considerations

This study was conducted with written approval from the University of Manitoba Health Research Ethics Board (HS19421) and the St. Boniface Hospital Research Review Committee (RRC/2016/1553).

## Results

### Patient characteristics

Data from 500 patients consisting of 2000 individual UHPLC-QTOF-MS assessments were available (two ESI-positive and two ESI-negative analyses from each study participant), which was merged with the database containing patient clinical covariates obtained from the Manitoba Tumour Bank. Four patients were excluded from the analysis due to the lack of consent to disclosure of clinical variables annotated to their samples; 11 patients were excluded from the analysis as they were identified as having provided more than one sample to the Manitoba Tumour Bank during the study period (duplicate patients). Loss of metabolomic data fidelity was detected in 10 patients and were excluded from the analysis.

Thus, data from 475 unique patients consisting of 241 lung cancer cases, and 234 cancer-free controls from which 1900 individual UHPLC-QTOF-MS assessments were conducted, were included in the final analysis. From these, a total of 2032 metabolic entities were detected in the ESI-positive mode, 1667 were detected in the ESI-negative mode, and 1529 entities were detected in both ESI modes. After filtering low prevalence entities, 676 metabolites remained of which 353 were detected in the ESI-positive mode and 323 from the ESI-negative mode which were used for further classification assessments.

The baseline clinical characteristics of the 475 included patients are shown in Table 1. Amongst the NSCLC cases, 177 (73%) had adenocarcinoma, and 64 (27%) had squamous cell carcinoma. For NSCLC cases, the final postoperative pathological staging (AJCC 6th ed.) was as follows: stage 1 (60%), stage 2 (21%), stage 3 (17%), and stage 4 (2%). NSCLC cases had a median age of 69 (range 49–88) versus 55 (range 20–89) for cancer-free controls (*p* < 0.001). Males comprised 46% of cases versus 29% of cancer-free controls, and the median body mass index was similar between cases (27.2, range 14.8 to 49.5) and controls (27.0, range 16.4 to 49.6). NSCLC cases had higher proportions with significant comorbidities including diabetes, cardiovascular disease, dyslipidemia, and hypertension. NSCLC cases had significant smoking history such that 27% were current smokers, 65% were previous smokers, and 8% were never smokers. By contrast, cancer-free controls had lower levels of smoking exposure such that 6% were current smokers, 22% were previous smokers, and 48% were never smokers.

**Table 1:** Baseline characteristics of the cohort

Variable	NSCLC cases (***n*** = 241)	Cancer-free controls (***n*** = 234)	***p***-value
**Age**			
Mean (range)	69 (49–88)	55 (20–89)	*p* < 0.001
**Sex**			
Male (%)	112 (46%)	69 (29%)	*p* < 0.001
Female (%)	129 (54%)	165 (71%)	
**Stage (AJCC 7th ed.)**			
I (%)	145 (60%)	N/A	-
II (%)	50 (21%)		
III (%)	41 (17%)		
IV (%)	5 (2%)		
**NSCLC type**			
Adenocarcinoma (%)	177 (73%)	N/A	-
Squamous cell carcinoma (%)	64 (27%)		
**Body mass index**			
Mean (range)	27.2 (14.8–49.5)	27.0 (16.4–49.6)	0.7
**Comorbidities**			
Diabetes (%)	55 (23%)	16 (7%)	< 0.001
COPD (%)	68 (28%)	17 (7.3%)	< 0.0001
Hypertension (%)	129 (54%)	44 (19%)	< 0.001
Dyslipidemia (%)	91 (38%)	35 (15%)	< 0.001
Cardiovascular disease (%)	72 (30%)	14 (6%)	< 0.001
**Smoking history**			
Current smoker (%)	65 (27%)	15 (6%)	< 0.001
Ex-smoker (%)	156 (65%)	51 (22%)	
Never smoker (%)	20 (8%)	48 (21%)	
Unknown (%)	0	120 (51%)	

### Cluster analysis

**Fig. 1:**
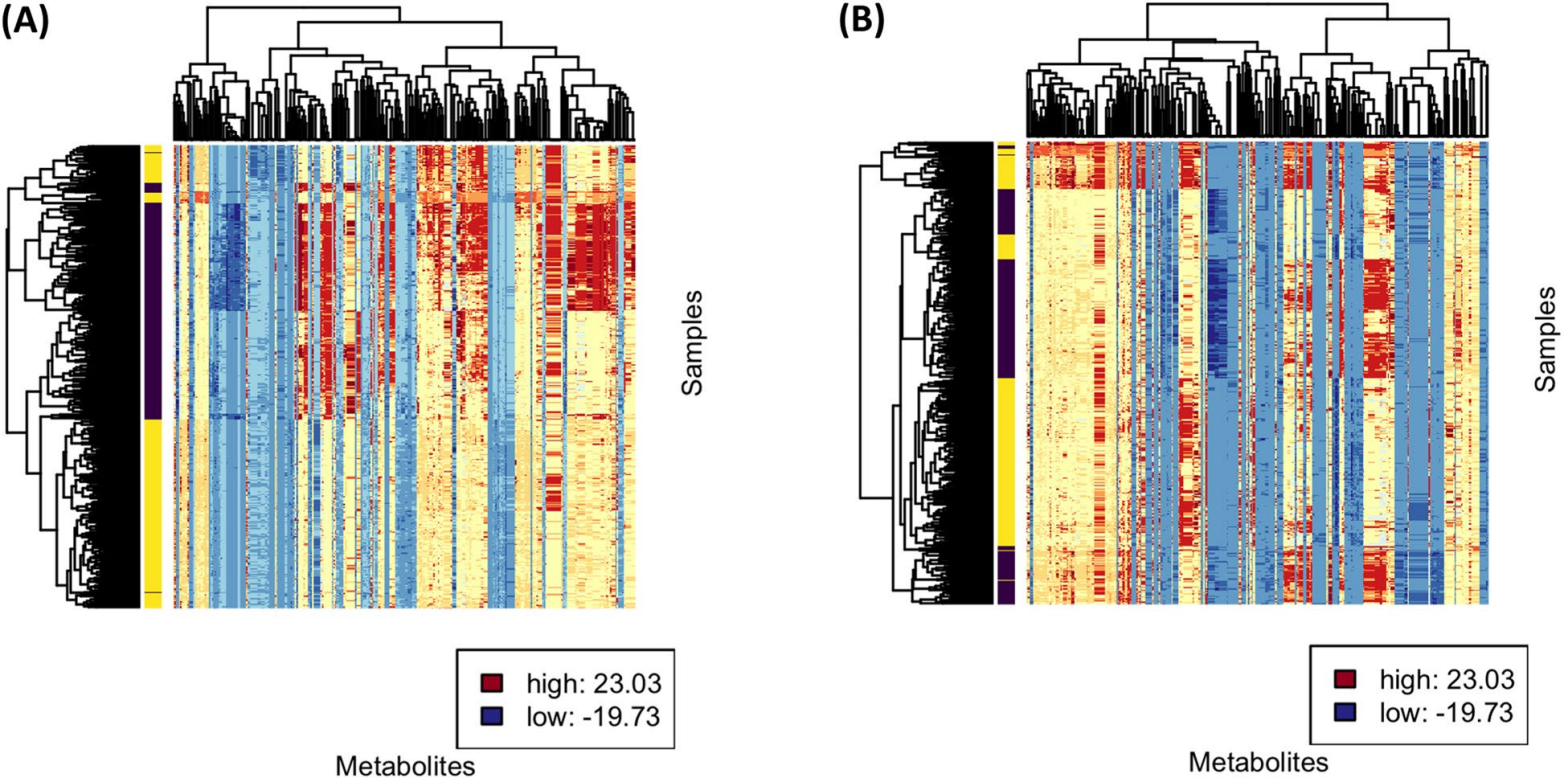
Hierarchical cluster analysis by correlations and heat map visualization for ESI-positive (**A**) and ESI-negative (**B**) metabolites. Color labels correspond to the 12 clusters used to identify representative metabolites. The interior of the heat map is colorized according to the Log2 normalized metabolite concentration, standardized to have means of zero and standard deviations of one: blue for low, yellow for average, and red for high. The samples are labeled yellow for cancer and purple for control

Metabolic entities were clustered based on their correlations to one another by their distance in vector space (1-correlation) using the complete linkage method, and samples were clustered based on Mahalanobis distance using the complete linkage in order to generate the cluster analysis and heat map visualization for ESI-positive (Fig. 1A) and ESI-negative (Fig. 1B) metabolites.

### Cluster-representative metabolites

The representative metabolite from each of the 12 identified clusters of metabolic entities is shown in Table 2. The complete list of metabolites found in each cluster is tabulated in a downloadable file (supplemental file 1). Amongst the ESI-positive entities, lipid metabolism (including phosphosphingolipids, glycerolipids), fatty acid metabolism, and steroids predominated as cluster-representative metabolites. Amongst ESI-negative entities, there was a greater variety of classes of metabolites which were observed as cluster-representative metabolites including the following: organic acids (ketoacids, carboxylic acids), steroids, fatty acyls, and hydroxyindol.

**Table 2:** Summary of cluster analysis representative metabolites

Cluster representative	ESI mode	Formula	m/z	Metabolite class	Function	Disease associations
Sphingosine 1-phosphate	+	C_18_H_38_NO_5_P	380.2533	Phosphosphingolipid	Cell survival, inflammatory response, lipid metabolism	Hepatocellular carcinoma [25], other cancers [26]
Pyridoxamine 5′-phosphate (vitamin B6)	+	C_8_H_13_N_2_O_5_P	249.0618	Pyridoxamines	Amino acid metabolism neurotransmitter biosynthesis, lipid metabolism	Ovarian cancer [27]
Sphinganine 1-phosphate	+	C_18_H_40_NO_5_P	382.2718	Phosphosphingolipid	Membrane stabilization	-
Calcidiol (25-hydroxyvitamin D)	+	C_27_H_44_O_2_	401.3432	Vitamin D and derivatives	Vitamin D precursor	Prostate, breast, and colorectal cancer survival [28]; conflicting data for lung cancer incidence [29, 30]
3-Methoxybenzenepropanoic acid	+	C_10_H_12_O_3_	181.0858	Phenylpropanoic acids	-	-
8-Hydroxyguanine	+	C_10_H_13_N_5_O_6_	168.0559	Purine derivative	Mutagenic base, marker of DNA damage	Lung and stomach cancer [31]
1b,3a,12a-Trihydroxy-5b-cholanoic acid	+	C_24_H_40_O_5_	409.3037	Steroids	Fat absorption and transport	-
Glycocholic acid	+	C_26_H_43_NO_6_	466.3250	Steroids	Fat emulsification, bile acid	Hepatocellular carcinoma [32], cholangiocarcinoma [33, 34], prostate cancer [35]
MG(0:0/18:1/0:0)	+	C_21_H_40_O_4_	357.2989	Glycerolipids	Lipid metabolism, lipid transport	-
2-Hydroxydecanedioic acid	+	C_10_H_18_O_5_	219.1263	Hydroxy acids	Cell membrane stabilizer, energy storage	Zellweger syndrome [36]
Gamma-carboxyethyl hydroxychroman (gamma-CEHC)	+	C_15_H_20_O_4_	249.1541	Benzopyrans	Vitamin E metabolism	Colorectal cancer [37]
Cholic acid glucuronide	−	C_30_H_48_O_11_	583.3146	Steroids	Cholesterol metabolism	-
Formaldehyde	−	CH_2_O	59.0137	Carbonyl compounds	Protein and nucleic acid metabolism	Leukemia [38], nasopharyngeal cancer [38]
17-Hydroxypregnenolone sulfate	−	C_21_H_32_O_6_S	411.1838	Steroids	Lipid metabolism, cell signaling	**-**
N_1_-Aceytylspermine	−	C_9_H_21_N_3_O	303.2322	Carboxylic acids	Cellular metabolism	**-**
Isodesmosine	−	C_24_H_40_N_5_O_8_	525.2811	Carboxylic acids	Elastin degradation	Liver cirrhosis [34, 39], cystic fibrosis [40, 41]
11-Beta-hydroxyandrosterone-3-glucuronide	−	C_25_H_38_O_9_	481.2450	Hydroxyindoles	Lipid metabolism	**-**
Lithocholic acid glycine conjugate	−	C_26_H_43_NO_4_	432.3120	Steroids	Fat excretion, absorption, and transport	**-**
3-Methyl-2-oxovaleric acid	−	C_6_H_10_O_3_	129.0549	Ketoacids	Amino acid metabolism	Maple syrup urine disease [42, 43]; colorectal cancer [44, 45]
18-Hydroxycortisol	−	C_21_H_30_O_6_	377.1993	Steroids	-	Primary aldosteronism [46, 47]
N(6)-Methyllysine	−	C_7_H_16_N_2_O_2_	159.1177	Carboxylic acid	Amino acid metabolism	**-**
Deoxycholic acid 3-glucuronide	−	C_30_H_48_O_10_	567.3370	Steroids	Fat emulsification	**-**
20-Carboxy-leukotriene B4	−	C_20_H_30_O_6_	411.1899	Fatty acyls	Lipid and drug metabolism	-
Pyroglutamic acid	−	C_5_H_7_NO_3_	128.0347	Carboxylic acid	Amino acid metabolism	NSCLC [48]

*Abbreviations*: *m/z* mass-to-charge ratio

### Principal component analysis

**Fig. 2:**
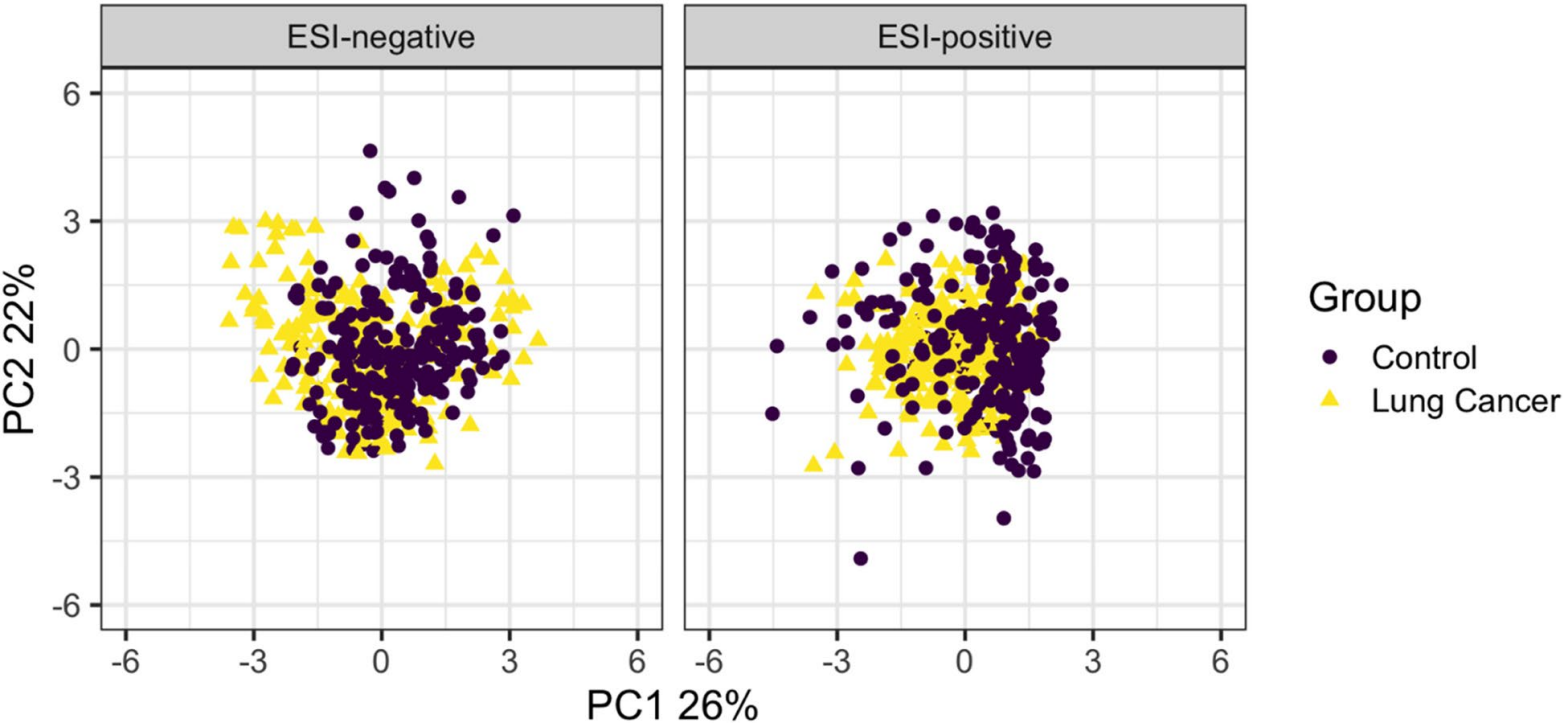
Principal component analyses of the cohort using the 12 cluster-representative metabolites from the ESI-positive and ESI-negative modes

Principal component analysis of the cohort using the 12 cluster-representative metabolites from the ESI-positive and ESI-negative modes demonstrated useful separation of the cohort by the first two principal components in each ESI mode (Fig. 2).

### Assessment of volcano plots

Assessment of the volcano plots (Fig. A1) reveals that most of the cluster-representative metabolites identified from cluster analyses are not outliers. A number of cluster-representative metabolites, however, were situated at or below the threshold of 0.5, indicative of smaller mean differential concentrations of the metabolites between cases and controls.

### Logistic regression modeling

**Fig. 3:**
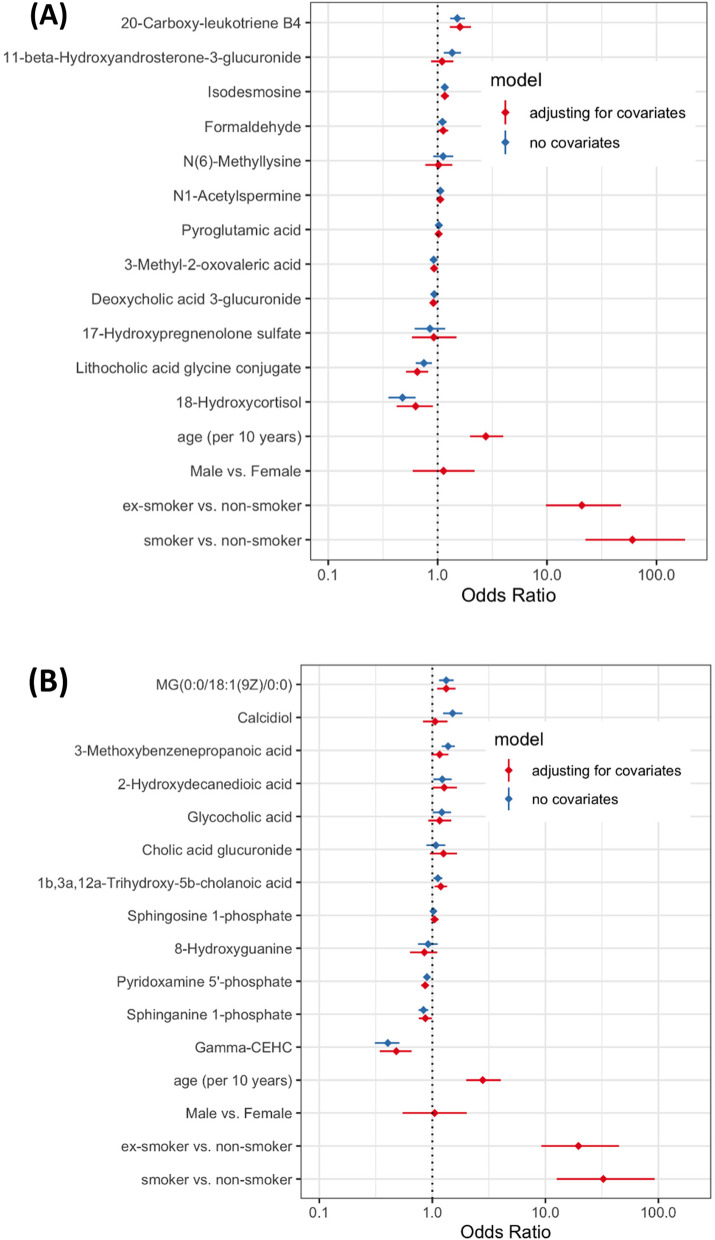
Forest plot of the distribution of odds ratios from the multivariable logistic regression analysis for cluster-representative metabolites with and without adjustment for covariates of age, sex, and smoking status metabolites for **A** ESI-positive and **B** ESI-negative analyses

Multivariable logistic regression analysis for the endpoint of NSCLC case status using the 12 cluster-representative metabolites revealed that a number of cluster-representative metabolites functioned as statistically significant predictors of NSCLC case status, while others did not (summarized as forest plots in Fig. 3A, B).

The ESI-positive class representative metabolites significantly associated with NSCLC case status in the logistic model of the metabolites only included the following: MG(0:0/18:1/0:0) (*OR* 1.33, 95% *CI* 1.15–1.55), calcidiol (*OR* 1.51, 95% *CI* 1.25–1.85), 3-methoxybenzenepropanoic acid (*OR* 1.38, 95% *CI* 1.21–1.59), glycocholic acid (*OR* 1.21, 95% *CI* 1.01–1.46), pyridoxamine 5′-phosphate (*OR* 0.90, 95% *CI* 0.85–0.95), sphinganine 1-phosphate (*OR* 0.84, 95% *CI* 0.75–0.92), gamma-CEHC (*OR* 0.40, 95% *CI* 0.31–0.51), and 1b,3a,12a-trihydroxy-5b-cholanoic acid (*OR* 1.12, 95% *CI* 1.03–1.23). The impact of adjustment for clinical covariates was substantial, whereby several cluster-representative metabolites lost statistical significance. The following ESI-positive cluster-representative metabolites conserved statistical significance after adjustment for clinical covariates: MG(0:0/18:1/0:0) (*OR* 1.33, 95% *CI* 1.10–1.60), pyridoxamine 5′-phosphate (*OR* 0.86, 95% *CI* 0.80–0.93), sphinganine 1-phosphate (*OR* 0.87, 95% *CI* 0.76–0.99), gamma-CEHC (*OR* 0.48, 95% *CI* 0.34–0.66), and 1b,3a,12a-trihydroxy-5b-cholanoic acid (*OR* 1.19, 95% *CI* 1.05–1.35).

The ESI-negative cluster-representative metabolites significantly associated with NSCLC case status in the multivariable logistic model of the metabolites only included the following: 20-carboxy-leukotriene B4 (*OR* 1.51, 95% *CI* 1.30–1.77), 11-beta-hydroxyandrosterone-3-glucuronide (*OR* 1.36, 95% *CI* 1.14–1.63), lithocholic acid glycine conjugate (*OR* 0.75, 95% *CI* 0.63–0.89), 18-hydroxycortisol (*OR* 0.48, 95% *CI* 0.35–0.63), formaldehyde (*OR* 1.11, 95% *CI* 1.01–1.21), isodesmosine (*OR* 1.16, 95% *CI* 1.09–1.24), 3-methyl-2-oxovaleric acid (*OR* 0.92, 95% *CI* 0.89–0.95), and deoxycholic acid 3-glucuronide (*OR* 0.93, 95% 0.89–0.97). The following ESI-negative cluster-representative metabolites maintained statistical significance after adjustment for clinical covariates: lithocholic acid glycine conjugate (*OR* 0.65, 95% *CI* 0.51–0.82), formaldehyde (*OR* 1.12, 95% *CI* 1.00–1.25), isodesmosine (*OR* 1.16, 95% *CI* 1.07–1.27), 18-hydroxycortisol (*OR* 0.63, 95% *CI* 0.42–0.91), 3-methyl-2-oxovaleric acid (*OR* 0.93, 95% *CI* 0.88–0.98), and deoxycholic acid 3-glucuronide (*OR* 0.91, 95% 0.85–0.98). The complete logistic regression analysis results are viewable in supplemental Tables 2 (ESI positive) and 3 (ESI negative).

### Classification performance of cluster-representative metabolites

**Fig. 4:**
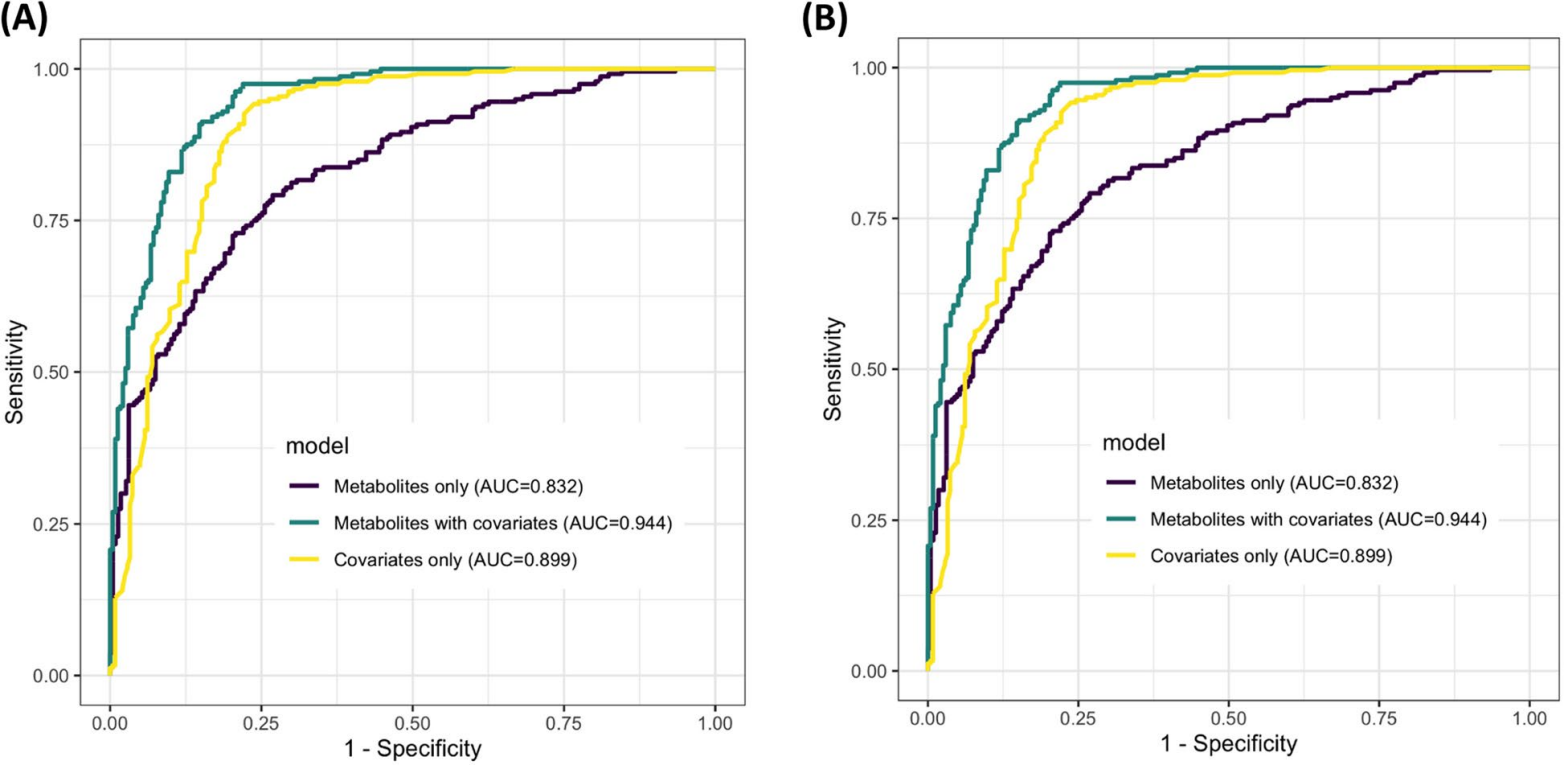
Receiver operator characteristic curves of cluster-representative metabolite logistic regression model with and without covariates (age, sex, smoking history) for ESI-positive (**A**) and ESI-negative (**B**) modes

The classification performance of the ESI-positive and ESI-negative models both with and without covariates is detailed in supplemental Table A4. Using the metabolites alone, the diagnostic accuracy of between 75 (ESI positive) and 82% (ESI negative) was observed. Diagnostic accuracy notably improved with the addition of clinical covariate variables (age, sex, smoking history) to 90% (ESI positive) and 94% (ESI negative). Receiver operator characteristic curves of the logistic regression models (Fig. 4) demonstrated an area under the curve (AUC) of 0.94 for both the ESI-positive and ESI-negative metabolites when clinical covariates were included in the model.

## Discussion

This study demonstrates that metabolomic analysis of plasma using UHPLC-QTOF-MS can potentially differentiate patients with clinical early-stage NSCLC from those who are lung cancer-free based on a wide variety of metabolites. To our knowledge, this study represents the largest cohort of patients with clinical early-stage NSCLC who have undergone non-targeted metabolomic analysis. We observed that plasma contains a number of classes of metabolites with potential classification utility, including phospholipids, fatty acids, steroids, fatty acyls, and amino acids. Cluster-representative metabolites of interest were identified including MG(0:0/18:1/0:0), 3-pyridoxamine 5′-phosphate, sphinganine 1-phosphate, gamma-CEHC, 1b,3a,12a-trihydroxy-5b-cholanoic acid from the ESI-positive mode, and lithocholic acid glycine conjugate, formaldehyde, isodesmosine, 18-hydroxycortisol, 20-carboxy-leukotriene B4, 3-methy-2-oxovaleric acid, and deoxycholic acid 3-glucuronide in the ESI-negative mode. Our findings suggest that these classes of metabolites and selected cluster-representative metabolites are worthy of further evaluation with targeted, quantitative metabolomic analyses for the development for the detection of early-stage NSCLC.

A number of the cluster-representative metabolites reported herein have been previously associated with oncogenesis. Alterations in fatty acid and lipid metabolism have been reported amongst studies assessing the serum or plasma [12, 13, 25] of lung cancer cases compared to controls. Sphinganine and sphingosine [12] overlap with several of the cluster-representative metabolites arising from this study, notably sphingosine-1-phosphate and sphinganine-1-phosphate. Phosphosphingolipids, including sphingosine 1-phosphate (S1P), are cell membrane-derived and have important roles in cell signaling, cell survival, inflammatory response, angiogenesis, and tumor growth [26]. S1P is implicated as a pro-tumorigenic factor which activates signaling pathways including Ras/ERK, PI3K/RAC, STAT3, and PLC which are associated with various cancers [27–30]. Steroid hormones including calcidiol (25-hydroxyvitamin D), the primary circulating form of vitamin D, and calcitriol (1,25-dihydroxyvitamin D), the primary active form of vitamin D, are implicated in the incidence [31] and prognosis [32] of a number of human malignancies including breast, colorectal, and prostate cancer. The association between vitamin D and NSCLC incidence and prognosis, however, remains controversial with conflicting directions of association reported [31] which may be partially explained by the complex interaction of effect modifiers including age, sex, and smoking status [33]. Curiously, in this study, we observed calcidiol to have utility as a classifier of NSCLC status, but its statistical significance in the logistic regression model disappeared when the clinical covariates were added to the model, not dissimilar to the aforementioned pathogenesis and pharmacoepidemiology studies. Of interest, a cluster-representative metabolite, 8-hydroxyguanine, is a mutagenic base which denotes hydroxyl-mediated DNA damage which is implicated in lung and gastric cancer oncogenesis in preclinical models [35]. This metabolite did not perform as well as other cluster-representative metabolites at predicting NSCLC status in this study but may merit further targeted evaluations in the future due to its potential link to oncogenesis.

Low-molecular-weight organic acids, including lactic acid, are known to be produced from cancers as a result of their altered glucose metabolism [36]. In this study, in the ESI-negative mode, a low-molecular-weight organic acid, pyroglutamic acid, was found to be a cluster-representative metabolite. Interestingly, pyroglutamic acid has been previously scrutinized for its discriminatory performance of serum from NSCLC versus matched controls in a panel of ten other candidate organic acids [37] and was found to have the best discrimination, with an AUC of 0.76. However, when compared with other cluster-representative metabolites from this study, pyroglutamic acid was outmatched in discriminatory capacity. This highlights the importance of starting metabolomic biomarker development processes with wide non-targeted approaches prior to narrowing down to further quantitative targeted approaches rather than the opposite.

The small overlap of NSCLC-specific altered metabolites reported in the literature as compared to our list of cluster-representative metabolites is likely a consequence of the heterogeneity of biofluids assessed, variations in pre/post-analytical laboratory procedures, and sample sizes utilized. Further differences may be explained by the choice of statistical methodology, driven by differences in analytical objectives, as well as preferred local best practices that have arisen in this rapidly growing field.

Exploratory analysis of the classification performance using representative metabolites from within each cluster of metabolites demonstrated classification accuracies in the range of 90%. With our experimental design planned to have a nearly 1:1 ratio of cases to controls, these results suggest further development work for this application of metabolomics is warranted. In this large cohort of patients with clinical early-stage NSCLC, we observed that age, sex, and smoking history, which are known risk factors for the development of NSCLC, significantly affected the performance of fitted predictive models. Of these, smoking status and age had particularly strong effects in the multivariable logistic regression models. The degree to which the clinical covariates influenced the classification characteristics of the logistic regression model mirrored the findings of Miyamoto et al., who found that the inclusion of age and sex improved the sensitivity and specificity of blood-based lung cancer metabolomic models [13]. These findings underline the need for future studies of metabolomic profiling for purposes of lung cancer detection to utilize comprehensively annotated biospecimens, including, at minimum, smoking history, age, and sex variables so to allow effective study design (e.g., stratified or matched sampling of cases and controls) as well as analytical methods such as multivariable adjustment [13] or propensity matching [16]. Furthermore, there are likely many other biologically plausible confounding clinical variables including ethnicity, body mass index, dietary, or lifestyle choices. Given the current paucity of published data assessing the impact of these additional covariates, any future metabolomics studies planned for this milieu should assess any potential impact of these variables on metabolomic profiles in order to improve the calibration of any future diagnostic models.

A limitation of this study is that specimen collection was not explicitly controlled for the prandial state. However, since many of our specimens were collected alongside other routine bloodwork which require fasting prior to sample collection, it is conceivable that a considerable proportion of samples were indeed collected in the fasting state. Unfortunately, since prandial status data were not recorded in the biorepository databases, it could not be verified for each patient. This issue may be common to biorepository specimens like those used in our study, as most biorepositories were conceived with genomic or transcriptomic studies in mind where the prandial state is of little importance as compared to metabolomic analyses. As a result, this study was unable to control for the effects of the prandial state. A literature search did not find any published data which explored the impact of the prandial state on lung cancer metabolomic profiles. Thus, future work in this milieu should be performed amongst patients who have undergone standardized pre-sampling preparations which would reduce the impact of the digestion of various foods on their metabolomic profiles used for lung cancer classification.

Metabolomic data sets are highly multidimensional, leading to an increased risk that key associations may go unnoticed as “noise” as opposed to “signal.” With this in mind, the next logical step in the development of a metabolomics profile aimed at the detection of early-stage lung cancer would be targeted metabolomic analyses using shorter, pre-specified lists of metabolites from biospecimens that are completely annotated with clinical covariate data and preferably collected in the fasting state. Based on the findings of this study, analyses targeting lipid, fatty acid, steroid, and amino acid entities such as some of the cluster-representative metabolites would be reasonable. By targeting specific regions of the metabolome, associations of individual metabolites with ES-NSCLC would be more accurately assessed and evaluated. These associations may be developed in clinical trials and in the setting of case-control trial design, for example, but will ultimately need to be studied and calibrated using cohort study designs where the prevalence of ES-NSCLC is a known truth. A potential source of such specimens and data may be found in image-based lung cancer screening programs which are already operational or are running in the context of a clinical trial.

## Conclusions

Global alterations in metabolism were found in the plasma of early-stage NSCLC cases compared to cancer-free controls. Further targeted analyses of specific metabolites and classes of metabolites using clinical covariate annotated biospecimens are warranted to refine this noninvasive approach to lung cancer detection.

## Supplementary Information

**Additional file 1: Supplemental Figure A1.** Volcano plots of the cluster-representative metabolites detected from the ESI positive and negative modes.

**Additional file 2: Supplemental Table A1.** Summary of Metabolomic Analysis Workflow. **Supplemental Table A2.** Estimated odds-ratios from muli-predictor logistic regression models based on representative ESI-positive metabolites, with and without adjustment for covariates. **Supplemental Table A3.** Estimated odds-ratios from muli-predictor logistic regression models based on representative ESI-negative metabolites, with and without adjustment for covariates. **Supplemental Table A4.** Classification performance of logistic regression models of cluster-representative metabolites both with and without adjustment for clinical confounding variables (Age, Sex, Smoking History) highlighting the importance of clinical covariates in metabolomic based assessments for lung cancer diagnosis Classification.

**Additional file 3: Supplemental File 1.** Table of all metabolites by cluster.

## Data Availability

All data generated and analyzed in this study are available by reasonable request of the corresponding author.

## Abbreviations

^1^H-NMR: ^1^H-nuclear magnetic resonance
CFC: Cancer-free controls
CIHR: Canadian Institutes of Health Research
CT: Computed tomography
ES-NSCLC: Early-stage non-small cell lung cancer
ESI: Electrospray ionization
MTB: Manitoba Tumour Bank
m/z: Mass-to-charge ratio
PCA: Principal component analysis
ROC: Receiver operator characteristic
S1P: Sphingosine 1-phosphate
UHPLC-QTOF-MS: Ultra-high-performance liquid chromatography/quadrupole time-of-flight mass spectrometry
