# Supplementary figures and images for: Data-driven identification of plasma metabolite clusters and metabolites of interest for potential detection of early-stage non-small cell lung cancer cases versus cancer-free controls
Source: Cancer Metab. 2022 Oct 12;10:16. doi: 10.1186/s40170-022-00294-9 (PMC9559833; doi:10.1186/s40170-022-00294-9)

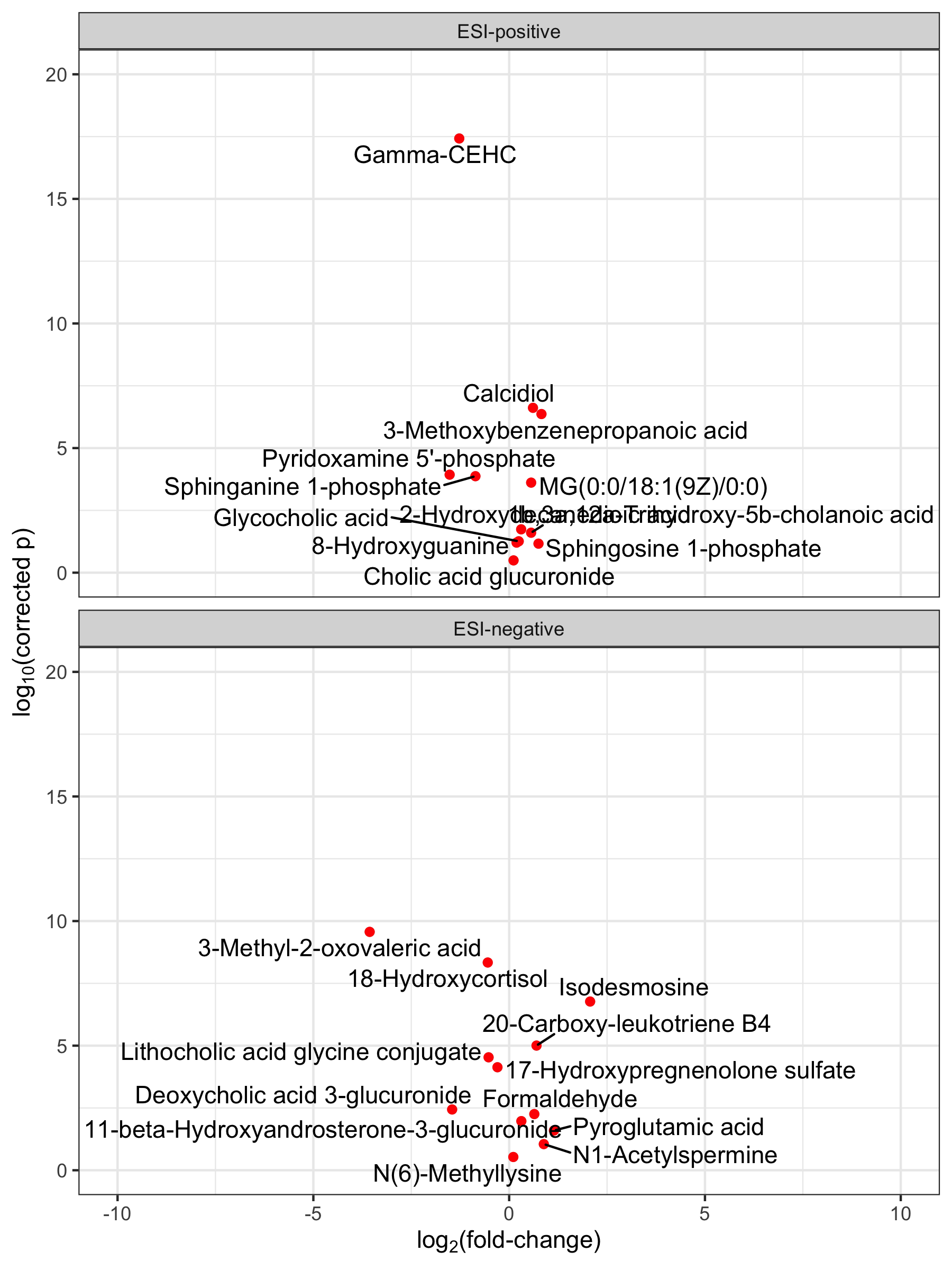

Supplement: Supplementary file 1 — Additional file 1: Supplemental Figure A1. Volcano plots of the cluster-representative metabolites detected from the ESI positive and negative modes. [file 40170_2022_294_MOESM1_ESM.tiff]
