# Supplementary material for: Data-driven identification of plasma metabolite clusters and metabolites of interest for potential detection of early-stage non-small cell lung cancer cases versus cancer-free controls
Source: Cancer Metab. 2022 Oct 12;10:16. doi: 10.1186/s40170-022-00294-9 (PMC9559833; doi:10.1186/s40170-022-00294-9)
Supplement: Supplementary file 2 — Additional file 2: Supplemental Table A1. Summary of Metabolomic Analysis Workflow. Supplemental Table A2. Estimated odds-ratios from muli-predictor logistic regression models based on representative ESI-positive metabolites, with and without adjustment for covariates. Supplemental Table A3. Estimated odds-ratios from muli-predictor logistic regression models based on representative ESI-negative metabolites, with and without adjustment for covariates. Supplemental Table A4. Classification performance of logistic regression models of cluster-representative metabolites both with and without adjustment for clinical confounding variables (Age, Sex, Smoking History) highlighting the importance of clinical covariates in metabolomic based assessments for lung cancer diagnosis Classification. [file 40170_2022_294_MOESM2_ESM.docx]

**Supplemental Tables:**

**Supplemental Table A1:** Summary of Metabolomic Analysis Workflow

**Step A: Metabolomics Analysis of Plasma Extracts (250 Early Stage NSCLC cases, 250 controls)**

1)Non-targeted analysis of plasma extracts by UHPLC-QTOF-MS in ESI positive and negative modes in duplicate for each sample

2) Molecular Feature Extraction (MFE) algorithm (Agilent Profinder Software V B.08, Agilent Technologies, USA) used to extract all detected compounds

3) Formula generation and Find-by-ion algorithm used to remove false +/- compounds

**Step B: Identification of Entities, Data Linkage, Data Cleaning, Data Filtration**

1) Metlin and Human Metabolome databases identified all known endogenous human metabolites from the detected entities

2) Linkage of metabolomic data to clinical covariate data (age, gender, smoking status) using R statistical software.

3) Data Cleaning: Loss of metabolomic data fidelity (10 patients dropped); Replicate Patients (11 patients dropped since they provided >1 sample during study period); Missing consent to disclose clinical variables (4 patients dropped)

4) Data Filtration: Candidate endogenous metabolites not detected >80% of samples were dropped from further analysis.

Results: 475 patients included in statistical analysis with 676 metabolites (353 ESI positive, 323 ESI negative)

**Step C: Statistical Analysis** **(R Statistical Software Package)**

1. Unsupervised hierarchical cluster analysis of metabolites and samples; generation of cluster analysis heat map by ESI mode
2. Principal Component Analysis (PCA). The metabolite with the strongest correlation to the first principle component of each cluster designated the “cluster representative metabolite”.
3. Volcano plots generated
4. Logistical regression analysis by ESI mode with cluster representative metabolites +/- clinical covariates as explanatory variables for the endpoint of NSCLC case status.
5. Forrest Plots generated of adjusted odds ratios of cluster representative metabolites used in the logistic regression analyses with and without adjustment for clinical covariates
6. Evaluation of classification performance of cluster representative metabolite assessing sensitivity, specificity, overall diagnostic accuracy; Receiver Operator Characteristic (ROC) Curves generated.

Supplemental Table A2: Estimated odds-ratios from muli-predictor logistic regression models based on representative ESI-positive metabolites, with and without adjustment for covariates.

|  | Unadjusted | | | Adjusted for Covariates | | |
| --- | --- | --- | --- | --- | --- | --- |
| Characteristic | OR^1^ | 95% CI^1^ | p-value | OR^1^ | 95% CI^1^ | p-value |
| 1b,3a,12a-Trihydroxy-5b-cholanoic acid | 1.12 | 1.03, 1.23 | 0.013 | 1.19 | 1.05, 1.35 | 0.008 |
| 2-Hydroxydecanedioic acid | 1.23 | 1.03, 1.48 | 0.030 | 1.27 | 1.01, 1.65 | 0.054 |
| 3-Methoxybenzenepropanoic acid | 1.38 | 1.21, 1.59 | <0.001 | 1.16 | 0.98, 1.39 | 0.091 |
| 8-Hydroxyguanine | 0.92 | 0.75, 1.11 | 0.4 | 0.85 | 0.64, 1.11 | 0.2 |
| Calcidiol | 1.51 | 1.25, 1.85 | <0.001 | 1.06 | 0.83, 1.36 | 0.7 |
| Cholic acid glucuronide | 1.08 | 0.89, 1.31 | 0.5 | 1.26 | 0.96, 1.65 | 0.10 |
| Gamma-CEHC | 0.40 | 0.31, 0.51 | <0.001 | 0.48 | 0.34, 0.66 | <0.001 |
| Glycocholic acid | 1.21 | 1.01, 1.46 | 0.039 | 1.16 | 0.92, 1.47 | 0.2 |
| MG(0:0/18:1(9Z)/0:0) | 1.33 | 1.15, 1.55 | <0.001 | 1.33 | 1.10, 1.60 | 0.003 |
| Pyridoxamine 5'-phosphate | 0.90 | 0.85, 0.95 | <0.001 | 0.86 | 0.80, 0.93 | <0.001 |
| Sphinganine 1-phosphate | 0.84 | 0.75, 0.92 | <0.001 | 0.87 | 0.76, 0.99 | 0.035 |
| Sphingosine 1-phosphate | 1.02 | 0.97, 1.08 | 0.4 | 1.05 | 0.97, 1.13 | 0.2 |
| gender |  |  |  | 1.05 | 0.55, 2.01 | 0.9 |
| age (per 10 years) |  |  |  | 2.79 | 2.00, 4.03 | <0.001 |
| smoke |  |  |  |  |  |  |
| non-smoker |  |  |  | — | — |  |
| ex-smoker |  |  |  | 19.6 | 9.22, 44.8 | <0.001 |
| smoker |  |  |  | 32.6 | 12.6, 92.8 | <0.001 |
| ^1^OR = Odds Ratio, CI = Confidence Interval | | | | | | |

Supplemental Table A3: Estimated odds-ratios from muli-predictor logistic regression models based on representative ESI-negative metabolites, with and without adjustment for covariates.

|  | Unadjusted | | | Adjusted for Covariates | | |
| --- | --- | --- | --- | --- | --- | --- |
| Characteristic | OR^1^ | 95% CI^1^ | p-value | OR^1^ | 95% CI^1^ | p-value |
| 11-beta-Hydroxyandrosterone-3-glucuronide | 1.36 | 1.14, 1.63 | <0.001 | 1.10 | 0.87, 1.39 | 0.4 |
| 17-Hydroxypregnenolone sulfate | 0.85 | 0.62, 1.17 | 0.3 | 0.92 | 0.58, 1.49 | 0.7 |
| 18-Hydroxycortisol | 0.48 | 0.35, 0.63 | <0.001 | 0.63 | 0.42, 0.91 | 0.016 |
| 20-Carboxy-leukotriene B4 | 1.51 | 1.30, 1.77 | <0.001 | 1.60 | 1.29, 2.02 | <0.001 |
| 3-Methyl-2-oxovaleric acid | 0.92 | 0.89, 0.95 | <0.001 | 0.93 | 0.88, 0.98 | 0.004 |
| Deoxycholic acid 3-glucuronide | 0.93 | 0.89, 0.97 | 0.003 | 0.91 | 0.85, 0.98 | 0.010 |
| Formaldehyde | 1.11 | 1.01, 1.21 | 0.028 | 1.12 | 1.00, 1.25 | 0.037 |
| Isodesmosine | 1.16 | 1.09, 1.24 | <0.001 | 1.16 | 1.07, 1.27 | <0.001 |
| Lithocholic acid glycine conjugate | 0.75 | 0.63, 0.89 | <0.001 | 0.65 | 0.51, 0.82 | <0.001 |
| N(6)-Methyllysine | 1.12 | 0.91, 1.39 | 0.3 | 1.02 | 0.77, 1.36 | 0.9 |
| N1-Acetylspermine | 1.06 | 1.01, 1.11 | 0.013 | 1.06 | 0.99, 1.13 | 0.095 |
| Pyroglutamic acid | 1.03 | 0.99, 1.07 | 0.2 | 1.02 | 0.96, 1.08 | 0.5 |
| gender |  |  |  | 1.13 | 0.59, 2.17 | 0.7 |
| age (per 10 years) |  |  |  | 2.76 | 1.97, 3.98 | <0.001 |
| smoke |  |  |  |  |  |  |
| non-smoker |  |  |  | — | — |  |
| ex-smoker |  |  |  | 20.8 | 9.77, 47.5 | <0.001 |
| smoker |  |  |  | 60.3 | 22.4, 182 | <0.001 |
| ^1^OR = Odds Ratio, CI = Confidence Interval | | | | | | |

**Supplemental Table A4: Classification performance of logistic regression models of cluster representative metabolites both with and without adjustment for clinical confounding variables (Age, smoking history, Sex) highlighting the importance of clinical covariates in metabolomic based assessments for lung cancer diagnosis**

| **Statistic** | **ESI Positive Cluster Rep Model** | | **ESI Negative Cluster Rep Model** | |
| --- | --- | --- | --- | --- |
|  | **With Covariates** | **No Covariates** | **With Covariate** | **No Covariates** |
| Sensitivity | 88% | 75% | 89% | 78% |
| Specificity | 85% | 75% | 84% | 74% |
| Diagnostic Accuracy | 91% | 75% | 94% | 82% |
